# Exploring Functional Products and Early-Life Dynamics of Gut Microbiota

**DOI:** 10.3390/nu16121823

**Published:** 2024-06-10

**Authors:** Ana B. Martínez-Martínez, Belen M. Lamban-Per, Maria Lezaun, Antonio Rezusta, Jose M. Arbones-Mainar

**Affiliations:** 1 Facultad de Ciencias de la Salud, Universidad de Zaragoza, 50009 Zaragoza, Spain; amarmar@unizar.es; 2 Instituto de Investigación Sanitaria Aragón, 50009 Zaragoza, Spain; arezusta@salud.aragon.es; 3 Department of Clinical Microbiology, Miguel Servet University Hospital, 50009 Zaragoza, Spain; bmlamban@salud.aragon.es (B.M.L.-P.); mlezaun@salud.aragon.es (M.L.); 4 Adipocyte and Fat Biology Laboratory (AdipoFat), Instituto Aragonés de Ciencias de la Salud (IACS), 50009 Zaragoza, Spain; 5 CIBER Fisiopatología Obesidad y Nutrición (CIBERObn), Instituto Salud Carlos III, 28029 Madrid, Spain

**Keywords:** early-life microbiota, gut functional products, dysbiosis biomarkers

## Abstract

Research on the microbiome has progressed from identifying specific microbial communities to exploring how these organisms produce and modify metabolites that impact a wide range of health conditions, including gastrointestinal, metabolic, autoimmune, and neurodegenerative diseases. This review provides an overview of the bacteria commonly found in the intestinal tract, focusing on their main functional outputs. We explore biomarkers that not only indicate a well-balanced microbiota but also potential dysbiosis, which could foreshadow susceptibility to future health conditions. Additionally, it discusses the establishment of the microbiota during the early years of life, examining factors such as gestational age at birth, type of delivery, antibiotic intake, and genetic and environmental influences. Through a comprehensive analysis of current research, this article aims to enhance our understanding of the microbiota’s foundational development and its long-term implications for health and disease management.

## 1. Introduction

The human microbiome refers to the collective assembly of microorganisms, their genes, and metabolic products present in and on the human body [1]. However, it encompasses a complex ecosystem that impacts host biology, including metabolic and immune functions. In contrast, “microbiota” specifically denotes the community of these microorganisms themselves, residing in particular environments such as the skin, mouth, and notably, the gastrointestinal tract. This distinction highlights that while the microbiota includes the organisms, the microbiome also comprises the genetic elements and bioactive compounds they produce, which contribute to the host’s physiological landscape.

Since the year 2000, the advancement of sequencing techniques, especially next-generation sequencing, has enabled the development of the field of metagenomics, which studies the genetic material of microorganisms. This allows us to understand the diversity and functions of the microbiota, not only in humans but also in any environment that can be inhabited by them [2].

In terms of biological relevance, this community of bacteria, viruses, fungi, and other unicellular organisms plays a key role in metabolism and has been linked to a wide range of diseases, from digestive disorders to neurological and metabolic conditions.

But for a deeper understanding of microbial activity, it is advisable to consider the metabolites produced by microorganisms, providing a snapshot of the biochemical activities taking place within the microbiome. By combining this approach with metagenomics, it is possible to link genetic potential with metabolic function.

On the other hand, culturomics, a term first coined in 2012, represents a novel approach aimed at enriching our understanding of microbiomes. This method diversifies culture conditions to closely replicate the natural environments of bacteria, facilitating the growth of previously unculturable species [3]. Unlike metagenomics, which relies on DNA sequencing to analyze microbial communities directly from environmental samples without the need for culturing, culturomics focuses on growing microorganisms in the lab under various conditions that mimic their natural habitats. By integrating culturomics and metagenomics, we can understand not just what the microbiome is capable of, but also what it is actively doing [4]. In this way, alterations in the microbiome due to dietary changes, probiotic use, antibiotics, or disease can be revealed. Understanding how these factors affect metabolic pathways, leading to changes in the host’s health, is opening new avenues for personalized therapies. It can also help in identifying biomarkers for diseases and inform the development of microbiome-based therapies by linking specific microbial genes with their metabolic products that impact health.

This approach enhances our grasp of the intricate interactions between microbiota and host health, offering the potential for more tailored and effective interventions to maintain or restore a healthy gut microbiome [5].

Yet, the spectrum of functions that the microbiota actively performs and contributes to human physiology is still largely untapped. These functions include the microbiota’s involvement in the modulation of the nervous system [6], its influence on aging processes [7], and its role in the development of autoimmune diseases beyond the gut [8]. Additionally, the interactions between dietary components, microbiota metabolites, and genetic expression in the host are complex and not yet fully mapped [9]. Furthermore, the role of the microbiota extends far beyond what was previously understood, marking a significant shift in the scientific perspective. Historically overlooked, the microbiota is now recognized as a pivotal player in a myriad of physiological processes and pathologies. Ongoing research continues to uncover its critical functions across various health conditions, demonstrating its profound impact. For instance, recent studies have even identified correlations between the breast microbiota and various tumor characteristics, as well as prognostic clinicopathologic features [10]. This expanding understanding underscores the microbiota’s integral role in health and disease, highlighting the necessity for further exploration and the potential it holds for future medical advancements.

In this review, we will explore the search for biomarkers, specifically focusing on functional products produced by the microbiota. These biomarkers include not only indicators of a healthy microbiota but also signs of potential dysbiosis, which could predict susceptibility to future diseases. By identifying these functional products, we aim to provide a clearer understanding of how the microbiota contributes to health and disease states. We will also focus on how the microbiota is formed from birth up to 3 years of age—a critical period during which the microbiota appears to reach a composition that remains relatively stable throughout adult life. Finally, we will examine the main factors influencing this establishment.

## 2. Gut Microbiota

It is considered that the majority of bacteria present in humans are concentrated in the gastrointestinal tract (GI), especially in the colon.

The human gut microbiome exhibits a foundational similarity among family members due to shared environmental factors and genetic backgrounds [11]. Despite these commonalities, individual microbiomes display distinct differences, particularly in the specific lineages and species present. This variation underscores the personalized nature of each person’s gut microbiota, which is composed of a diverse array of bacteria ranging from 500 to 1000 species. The vast number of bacterial cells in the human gut, slightly outnumbering human cells, and a genomic presence that is 100 times larger than the human genome—approximately 38 trillion cells and 2 million bacterial genes—highlight the complex interplay of shared and unique microbial characteristics within families [12,13].

**Figure 1:**
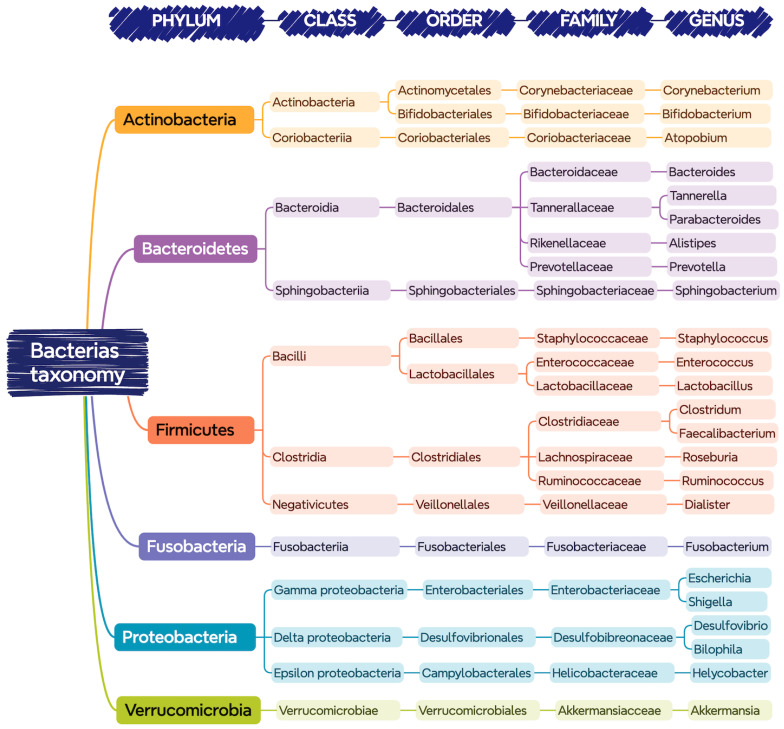
Bacteria present in the gastrointestinal tract classified according to their taxonomy (phylum, class, order, family, and genus).

Figure 1 shows the bacteria that are commonly found in the gastrointestinal tract of a healthy person, classified according to their phylum, class, order, family, and genus.

Despite the indicated diversity, it seems that there is an optimal balance of this core of bacteria for each person, in which those belonging to the phyla Bacteroidetes and Firmicutes are predominant [14].

Next, we will describe main functions associated with each of the mentioned phyla in the gut. It is worth considering that there is variability in the specific functions, with the potential for distinct or complementary roles in the colon ecosystem at the family or genus level within each phylum.

### 2.1. Actinobacteria

This phylum is divided into six classes: *Actinobacteria*, *Acidimicrobiia*, *Coriobacteriia*, *Nitriliruptoria*, *Rubrobacteria*, and *Thermoleophilia*. They are primarily Gram-positive filamentous bacteria, most of them aerobic and saprophytic in nature [15]. They have the ability to compete with pathogens for nutrients and adhesion sites on the intestinal mucosa, producing antimicrobial substances that inhibit the growth of harmful bacteria, especially the genus *Bifidobacterium*, a lactic acid producer, considered as a positive stimulus for intestinal health and immune system development and widely used as a probiotic [16].

Moreover, like *Firmicutes*, *Actinobacteria* break down fibers and complex carbohydrates that are not digested in the small intestine, producing SCFAs [17]. In addition, they can synthesize certain B complex vitamins, such as biotin (B7) and folic acid (B9), important for cellular metabolism and DNA production [18].

### 2.2. Bacteroidetes

This phylum is comprised of six classes of Gram-negative bacteria that are non-spore-forming, non-motile, and mostly anaerobic. The *Bacteroidetes* break down complex polysaccharides and dietary fiber ingested through the diet. As a result of their metabolism, a wide range of metabolites is produced, such as isopedopeptins, related to antibiotic resistance; pigments, with antioxidant function; or short linear peptides, many of them with immunogenic characteristics [19].

### 2.3. Firmicutes

The phylum *Firmicutes* includes Gram-positive bacteria, many of which are spore-forming. They can be obligate anaerobes, as well as aerobes or facultative anaerobes. Bacteria belonging to this phylum can adopt different shapes, such as cocci, bacilli, or filaments. Additionally, it includes genera beneficial to health as well as those with potential pathogenic effects [20].

Gut *Firmicutes* are known to contain numerous genes that facilitate the fermentation of dietary fiber. Additionally, they may interact with the intestinal mucosa, contributing to the maintenance of homeostasis. [21]. As a result of their metabolism, they generate short-chain fatty acids (SCFAs), including acetate, propionate, butyrate, valerate and isovalerate, among others [22].

They are also capable of producing vitamin K2, important for blood clotting and bone health, as well as several B complex vitamins, including riboflavin (B2), essential for energy metabolism; cobalamin (B12), necessary for nerve function and blood formation; folic acid (B9), crucial for DNA synthesis and cell division; and biotin (B7), involved in the metabolism of lipids, proteins, and carbohydrates [23].

### 2.4. Fusobacteria

*Fusobacteria* are generally Gram-negative bacilli, predominantly anaerobes, considered opportunistic pathogens in humans. Some exhibit a fusiform morphology, hence their name [24].

Their role in the gastrointestinal tract is not fully defined. It seems they interact with the rest of the microbiota’s microorganisms, activating the inflammatory response designed to protect against pathogens that promote tumor growth. In fact, a higher abundance of these microorganisms has been shown in the presence of colorectal cancer (CRC) [25,26]. Tumors characterized by a high level of inflammation, such as those enriched with granulocytes and *Fusobacterium*, have the worst prognosis [27]. Furthermore, resistance to chemotherapy is a major cause of tumor recurrence and poor prognosis in patients with CRC. Studies have reported a higher abundance of *Fusobacterium nucleatum* in the CRC tissues of patients with post-chemotherapy recurrence compared to those without recurrence, suggesting that *Fusobacteria* might play a role in chemoresistance. Additionally, *Fusobacterium nucleatum* has been shown to inhibit the recruitment of anti-cancer tumor-infiltrating T cells, further complicating therapeutic interventions [28].

### 2.5. Proteobacteria

This phylum of Gram-negative bacteria is highly diverse, consisting of more than 200 genera that include both beneficial and inherently pathogenic bacteria. Although *Proteobacteria* are less abundant in the intestine compared to the phyla *Bacteroidetes* and *Firmicutes*, they exhibit extensive metabolic diversity. This allows them to participate in a variety of biochemical processes within the intestinal ecosystem and to metabolize a broad range of organic compounds, thus contributing to the complexity and dynamism of the intestinal microbiome. While beneficial *Proteobacteria* contribute to nutrient processing and support the immune system, certain genera such as *Shigella* spp. or *Vibrio cholerae* are primarily recognized for their pathogenic potential. In some studies, an increase in the proportion of *Proteobacteria* in the intestine has been associated with states of dysbiosis, potentially serving as an indicator of inflammatory bowel disease, obesity, and other related diseases [29,30].

### 2.6. Verrucomicrobia

These are Gram-negative bacteria that present a diversity of shapes such as cocci, bacilli, and spirals. Some of them have a type of appendage that gives them a warty appearance—the reason for their name.

Within this phylum, the genus *Akkermansia* has the capacity to degrade mucin, a component of intestinal mucus. This activity aids in the renewal and maintenance of the mucus layer’s thickness, essential for preventing the passage of harmful substances and microorganisms from the intestine into the bloodstream. Indeed, its abundance has been inversely associated with people with cardiovascular diseases and certain types of cancer, suggesting a potential probiotic function [31]. Specifically, *Akkermansia muciniphila* helps preserve a healthy gut barrier, which in turn regulates immunity and curbs the onset of inflammation. This inflammation is a fundamental factor in the development of many diseases [32].

## 3. Functional Products of the Intestinal Microbiota

By functional products of the microbiota, we refer to small bioactive molecules resulting from the metabolic activity of intestinal bacteria. These substances have both local and systemic effects, significantly contributing to metabolism regulation, the maintenance of intestinal barrier integrity, the modulation of the immune system, and protection against pathogens.

The complex interaction between these functional products and the host organism underscores the importance of a balanced microbiome for overall well-being and highlights the therapeutic potential of manipulating the microbiota to prevent and treat diseases [33].

**Figure 2:**
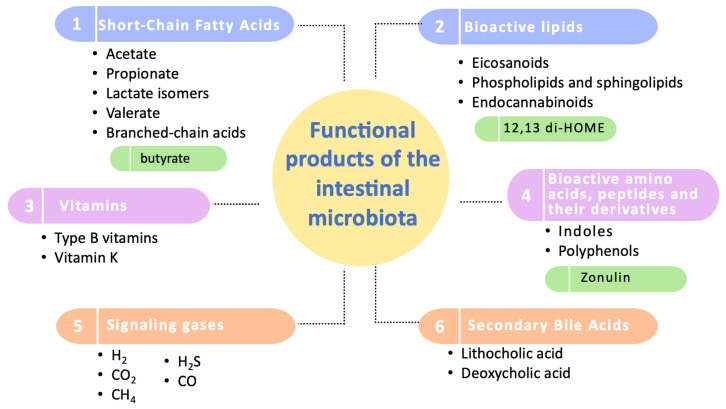
Categorization of the functional products of the intestinal microbiota. This diagram illustrates the primary categories of functional products produced by the intestinal microbiota, highlighting the most extensively studied compounds within each category. Key biomarkers of significant influence on human health are emphasized in green. It should be noted that zonulin, although included, is primarily produced by human cells and not by the microbiota.

Regarding these studied functional products, they have been categorized into six groups: short-chain fatty acids, bioactive lipids, vitamins, amino acids and bioactive peptides, signaling gases, and secondary bile acids (Figure 2).

### 3.1. Short-Chain Fatty Acids (SCFAs)

As previously indicated, SCFAs are produced by anaerobic bacteria present in the intestine through the bacterial fermentation of fiber and other non-digestible carbohydrates. These dietary compounds, such as resistant starch, fructooligosaccharides (FOSs), and galactooligosaccharides (GOSs), escape digestion and absorption in the small intestine, reaching the colon, where they are utilized for their own metabolism by these bacteria [22].

SCFAs are, by definition, organic molecules composed of a chain of less than six carbon atoms. Thus, they include acetate (C2), propionate (C3), and butyrate (C4). Their short carbon chain endows them with unique physicochemical properties—they are water-soluble and play crucial roles in various biological processes in the human body [34].

In the intestine, among all SCFAs present, acetate is the most abundant (60%), while propionate and butyrate are found at 20%. Additionally, lactate isomers, valerate, and branched-chain SCFAs such as isobutyrate and isovalerate are detected, but their levels are noticeably lower than the rest [35].

Despite the abundance of acetate, the role of butyrate stands out, as it is considered the main food source for colonocytes [36]—the cells that inhabit the colon, whose main functions are:

- Maintain the water and electrolyte balance of the body, being responsible for the absorption of water and electrolytes (like sodium and chloride) from waste materials passing through the colon and for the formation of feces of adequate consistency.
- Act as a physical and biochemical barrier that protects against the invasion of pathogens and the entry of toxic substances from the intestinal lumen into the bloodstream. This barrier function is maintained by tight junctions between epithelial cells, as well as by mucus production by neighboring goblet cells.
- Contribute to the regulation of the immune response in the intestine. Through interaction with immune cells and the production of cytokines and chemokines, colonocytes help maintain immunological tolerance and prevent excessive inflammatory responses.
- Maintain the integrity of the intestinal lining against daily wear and/or after injuries or inflammation.

SCFAs can induce both differentiation and the apoptosis of colonocytes, potentially playing a fundamental role in the prevention of colon cancer [37].

Regarding the specific functions of these fatty acids, SCFAs, mainly butyrate, stimulate the concentration of tight junctions by activating genes that encode these proteins. Additionally, they stimulate the formation of Mucin 2, essential for maintaining the mucus layer of the intestinal epithelium [38]. These actions help preserve the permeability and integrity of the intestinal barrier.

Butyrate also intervenes in the modulation of oxidative stress, reducing DNA damage induced by H_2_O_2_ by restoring the levels of the antioxidant glutathione [39].

Furthermore, they are involved in various physiological processes of the nervous system. For example, propionate can enter the portal circulation, activating the free fatty acid receptor 3 (FFAR3) on the surface of afferent periportal neurons and inducing intestinal gluconeogenesis. SCFAs also regulate the inhibition of histone deacetylase (HDAC), implicated in several neurological diseases like depression, schizophrenia, and Alzheimer’s [40]. Additionally, they participate in systemic neuroinflammation and serotonin biosynthesis, affecting emotions, cognition, and mental disorders [41].

In animal models, a higher presence of SCFAs, especially acetate, has been linked to reduced body weight and decreased appetite [42].

In the cardiovascular area, high levels of butyrate and propionate have been associated with a decrease in blood pressure and levels of plasminogen activator inhibitor-1 (PAI-1), a pro-thrombotic factor [43].

Lastly, the functions of SCFAs regarding immune function are being studied [44]. They seem to act directly on neutrophils, decreasing the production of reactive oxygen species (ROS) and myeloperoxidase (MPO), as well as favoring their apoptosis. Moreover, they reduce the chemotaxis of inflammatory cells by decreasing the expression of MCP-1 (monocyte chemoattractant protein-1), VCAM1 (vascular cell adhesion molecule-1), and chemokines. In this way, they prevent exacerbated inflammatory reactions. Regarding adaptive immunity, they could increase the number and activity of Treg cells, inhibit CD4+ T lymphocytes, decrease NF-κB signaling, and increase IL-10 and other anti-inflammatory cytokines [45], favoring an anti-inflammatory environment. Thus, they are considered as a protective factor against pathologies associated with excessive inflammation such as inflammatory bowel diseases (IBDs).

### 3.2. Bioactive Lipids

Besides short-chain fatty acids (SCFAs), eicosanoids, phospholipids, sphingolipids and endocannabinoids have important functions in regulating the immune system, inflammation, and metabolism [46].

Eicosanoids, primarily derived from arachidonic acid (AA), a polyunsaturated ω-6 fat, include prostaglandins (PGs), leukotrienes, thromboxanes, and lipoxins. These entities play a role in various physiological and homeostatic functions such as regulating vascular tone, controlling platelet aggregation, and managing pain perception. However, they are primarily recognized for their involvement in immunity and inflammation, where they serve as initiators for initiating the inflammatory response. [47]. In fact, when the integrity of the intestinal barrier is lost, some PGs, lipoxins, and leukotrienes amplify the signal of certain pro-inflammatory cytokines (TNF-α, IL-1β, and Il-6 among others), exacerbating the inflammatory response and being associated with chronic inflammatory diseases [48]. As an example, regarding inflammatory bowel disease (IBD), the study by Gobbetti et al. [49] demonstrated that a systemic treatment with ω-3 docosapentaenoic acid DPA-derived protectin D1 and ω-3 DPA-derived resolvin D5 protected against colitis and intestinal ischemia/reperfusion-induced inflammation in mice.

Additionally, a molecule that has been gaining importance is 12-13-diHOME (12,13-dihydroxy-9Z-octadecenoic acid). It is an oxylipin, a product of the metabolism of linoleic acid, a type of omega-6 fatty acid, formed through mono- or dioxygenase action. Gut bacteria, such as *Enterococcus faecalis* and *Bifidobacterium* spp., encode epoxide hydrolase enzymes capable of generating 12,13-diHOME [50]. This compound can act on dendritic cells, promoting a Th2-type inflammatory response, with its elevation being related to the development of allergic processes. On the other hand, an increase in this molecule in plasma has been found in response to physical exercise and exposure to cold, stimulating the conversion of brown adipose tissue (BAT) into white adipose tissue (WAT); thus, it is considered protective against obesity [51].

Regarding phospholipids and sphingolipids, these are fundamental components of cell membranes, and their functions are thus primarily related to inflammation, vesicular trafficking, and endocytosis [52]. A study conducted in mice by Brown et al. [53] demonstrated that patients with inflammatory bowel disease exhibit lower levels of Bacteroides-derived sphingolipids in their feces and elevated levels of host sphingolipids, which inversely correlate with the abundance of Bacteroidetes. These findings underscore their role as targets for the treatment of diseases characterized by chronic inflammatory processes.

Another type of lipid produced by the gut microbiota are the endocannabinoids, which are not exclusive to microbial action and are present in many organs and tissues. The most studied of these ligands are N-arachidonoylethanolamide (AEA), 2-arachidonoylglycerol (2-AG), O-arachidonoylethanolamine (EA), N-oleoylethanolamine (OEA), and N-palmitoylethanolamine (PEA) [54]. They play a role in both the innate and adaptive immune responses, exerting a potent anti-inflammatory effect. Among them, PEA has been associated with a protective effect in patients suffering from Alzheimer’s and Parkinson’s diseases. Furthermore, it is considered a fat sensor by mediating the response to high-fat diets and regulating thermogenic processes through the activation of PPAR-α [55]. Additionally, endocannabinoids like AEA and 2-AG are known to influence appetite regulation, primarily through their interaction with cannabinoid receptor-1 (CNR1). These interactions tend to stimulate hunger and increase food intake, whereas OEA appears to inhibit hunger [56].

### 3.3. Vitamins

Bacterial species such as *Bacteroides* spp., *Bifidobacterium* spp., and *Enterococcus* spp. are capable of synthesizing several vitamins such as type B vitamins (such as thiamine, folate, riboflavin, pantothenic acid) or vitamin K. These vitamins are involved in several essential functions such as blood coagulation, DNA synthesis, or energy metabolism. Interestingly, the molecular structure of vitamins synthesized by bacteria occasionally diverges from that of identical components consumed in the diet, influencing, for instance, the necessity of different transporters for their absorption [57].

Kang et al. [58] conducted a study using the *Caenorhabditis elegans* model, demonstrating that vitamin B12-producing bacteria that colonize the intestine can reduce cholinergic signaling in the nervous system through a process of rewiring the methionine cycle in the intestine. The vitamin B12 produced reduces cholinergic signaling by limiting the availability of free choline, which is needed by neurons for the synthesis of acetylcholine.

It is noteworthy that other gut bacteria, such as butyrate-producing bacteria from the Firmicutes genus, require vitamins for their growth, which can come from both the diet and their production by other bacteria in the colon [59]. This fact suggests the need to supplement prototrophic bacteria with these vitamins to stimulate the proliferation of butyrate-producing bacteria. Moreover, it highlights that fermentable fiber is not the only nutrient that affects microbial composition [60].

### 3.4. Bioactive Amino Acids, Peptides and Their Derivatives

It has been established that the colonic microbiota exhibits proteolytic power, particularly the *Bacteroides*, which are capable of breaking down both endogenous and exogenous proteins into peptides, amino acids, and derivatives [61,62].

Bioactive peptides are recognized for their wide range of beneficial activities, such as antimicrobial, antioxidant, antihypertensive, immunomodulatory, hypocholesterolemic, opiate-like, mineral-binding, and antithrombotic properties [63,64,65,66]. Highlighting the practical impact of such peptides, Liu et al. [66] conducted studies demonstrating that the fermentation of the peptide fraction from *Dendrobium aphyllum* with human fecal microbiota not only released antioxidant peptides but also promoted the proliferation of intestinal bacteria. 

Zonulin, an enterotoxin secreted by intestinal epithelial cells in response to dietary or microbiota stimuli, is garnering significant interest for its role in regulating the competency and function of tight junctions (TJs) in the intestinal epithelium. Elevated levels of zonulin have been linked to enhanced intestinal permeability, thereby increasing susceptibility to potentially harmful substances entering the bloodstream. This increased permeability has been associated with various autoimmune diseases, such as celiac disease and type 1 diabetes, highlighting its potent regulatory role in intestinal barrier function [67,68].

Additionally, gut microbiota can metabolize tryptophan to produce indole, which can subsequently be transformed into other metabolites such as indole-3-acetic acid (IAA), indolepropionic acid, and indole-3-lactate [69]. These compounds have been proposed as therapeutic targets as they activate nuclear receptors and regulate intestinal hormones, thereby aiding in the maintenance of intestinal homeostasis and positively impacting liver metabolism and immune response [70,71].

Polyphenols are also being studied for their potent anti-inflammatory and antioxidant properties. The intestinal microbiota can produce some of these compounds through the endogenous metabolism of aromatic amino acids. Additionally, more complex polyphenols derived from plant sources enter the intestinal tract through dietary intake and are subsequently metabolized in the colon by the resident microbiota. This fermentation process results in the production of a wide range of metabolites, which play a crucial role in mitigating chronic diseases [72,73,74,75].

### 3.5. Signaling Gases

Among the products resulting from the fermentation of carbohydrates, proteins, and other compounds by intestinal bacteria, gases are also produced. More than 99% of intestinal gas is composed of hydrogen (H_2_), carbon dioxide (CO_2_), and methane (CH_4_). The remainder includes other gases and volatile elements such as hydrogen sulfide (H_2_S) and carbon monoxide (CO) [72]. Bacteroides and Clostridium species are considered major producers of these microbial gases [76].

These gases are not merely byproducts; they can also perform significant functions. Some possess antimicrobial properties, can act as signaling molecules or neurotransmitters, stimulate intestinal transit, protect against oxidative damage, and even influence the immune response [77].

In their review, Ichikaea et al. [78] emphasize the benefits of hydrogen-producing bacteria, noting that a reduction in the number of these bacteria has been linked to dysbiosis. They highlight the bidirectional gut–brain relationship where hydrogen molecules play a protective role both in the gut against hepatitis induced by concanavalin A and in the brain, where they help mitigate neuronal changes associated with conditions such as depression and dementia.

### 3.6. Secondary Bile Acids

Bile acids are synthesized in the liver from cholesterol, secreted into the intestine to function as detergents, and emulsify dietary lipids and cholesterol to facilitate their absorption. Once their function is completed, they are reabsorbed in the ileum and circulate back to the liver, where they are re-secreted to establish their enterohepatic circulation [79].

Bile acids are not synthesized by the intestinal microbiota. Nonetheless, the microbiota plays a vital role in metabolizing and transforming these bile acids after their secretion into the intestine. Present bacteria can modify these primary bile acids, converting them into secondary bile acids through processes such as dihydroxylation; thus, they are considered to be metabolites derived from intestinal microbial activity [80].

These transformations affect the solubility and reabsorption capacity of bile acids, as well as their biological functions, resulting in them being considered more than mere detergents and extensively studied for their significant role in cellular signaling [81].

Therefore, the composition and activity of the intestinal microbiota can have a significant impact on the bile acid profile in the body and, by extension, on various physiological and pathological functions, including the regulation of lipid metabolism, inflammation, and resistance to metabolic and hepatic diseases. In fact, it has been described in murine models how secondary bile acids, such as lithocholic acid and deoxycholic acid, reduce the risk of hypertension. These acids act as agonists for farnesoid X receptors and TGR5, leading to a reduction in inflammation and fibrosis [82].

## 4. Development of the Microbiota in the First Years of Life

The first 1000 days of life, spanning from conception to approximately three years of age, are considered a critical window for the development of the intestinal microbiota. The composition and diversity of the microbiota established during this period can have lasting effects on an individual’s health, including predisposition to diseases such as obesity, allergies, autoimmune diseases, and metabolic disorders [83].

**Figure 3:**
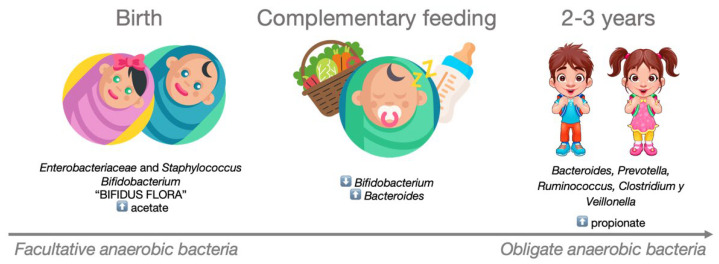
Development of the microbiota in the early years of life. During the first two years of life, the composition of a child’s intestinal microbiota evolves from being dominated by facultative anaerobic bacteria to predominantly obligate anaerobic bacteria.

After birth, the infant’s intestinal microbiota is initially dominated by *Enterobacteriaceae* and *Staphylococcus* spp., which are subsequently replaced by *Bifidobacterium* spp. and other lactic acid-producing bacteria. This composition, known as ‘Bifidus flora’ persists until complementary foods are introduced into the baby’s diet. The weaning process triggers a progressive increase in the presence of *Bacteroides*, leading to the displacement of *Bifidobacterium*. By the age of 2–3 years, the microbial community begins to resemble that of adults, primarily composed of species such as *Bacteroides* spp, *Prevotella* spp, *Ruminococcus* spp., *Clostridium* spp., and *Veillonella* spp. Accompanying these microbiota changes are shifts in the production and diversity of SCFAs. During the early stages of life, the concentration of acetate is high, primarily produced by the *Bifidobacterium* strains that characterize the infant intestinal microbiota. These strains metabolize human milk oligosaccharides as an energy source [84]. Following the cessation of breastfeeding and the introduction of complementary foods, intestinal levels of propionate increase, driven by a larger proportion of *Firmicutes*, particularly from the *Clostridia* class. This entire process is depicted in Figure 3.

### 4.1. Initial Colonization

Until recently, it was thought that the bacterial colonization of the intestinal tract began during childbirth and was a dynamic process. However, recent studies suggest that such colonization begins in the uterine environment [83] and does not end after the transfer of intestinal and vaginal microbiota following birth [85]. Breast milk is also considered a source of microbiota transfer to the newborn [86,87,88]. In fact, the enteric–mammary axis has been described [89], meaning that microorganisms and/or their functional products can be carried from the maternal intestine to the mammary glands, directly influencing the initial colonization of the newborn.

During the first years of life, the microbial population inhabiting the gastrointestinal tract includes facultative anaerobic bacteria. *Actinobacteria*, especially *Bifidobacterium*, are among the first colonizers of the intestinal tract.

**Figure 4:**
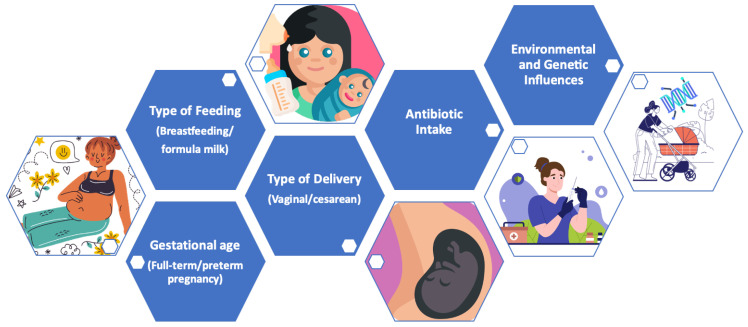
Main modulating factors of the microbiota in early life.

Below, the main modulating factors of the microbiota during the early years of life are indicated (Figure 4).

### 4.2. Gestational Age

It should be noted that babies born at full term reach an adult microbiota more quickly, compared with preterm infants, which are characterized by a lower diversity of the intestinal microbiota compared to full-term babies. In fact, differences in the vaginal microbiota have been noted between women who gave birth to full-term babies compared to those who had preterm babies, pointing out that mothers of preterm children had dysbiosis during pregnancy [90].

### 4.3. Type of Delivery

Childbirth plays a crucial role in establishing the initial colonization of the newborn’s intestinal microbiota [91,92,93]. Previously, it was believed that the fetal gastrointestinal system was sterile. However, recent research indicates that the transfer of commensal bacteria from mother to child might occur before birth. Studies have found small quantities of bacteria in initial meconium samples from newborns delivered at full term and in good health [94].

In infants born through vaginal delivery, *Lactobacillus* spp. is found on their skin, in their mouths, and intestines, being one of the most abundant microorganisms in the maternal vaginal flora. Besides vaginal bacteria, uterine and placental bacteria swept along to the newborn seem to foster tolerance towards microorganisms that promote postnatal well-being.

Conversely, infants born via cesarean section possess a microbiota similar to maternal skin, with *Staphylococcus* spp. dominating, followed by *Propionibacterium* spp. and Corynebacterium. Cesarean section has been associated with a lesser abundance and diversity of the phyla *Actinobacteria* and *Bacteroidetes* and a greater abundance and diversity of the phylum *Firmicutes* from birth to 3 months of life [95]. At the colonization level, the genera *Bifidobacterium* and *Bacteroides* seem to be significantly more common in vaginally delivered infants compared to those delivered by cesarean section, which are more colonized by the genera *Clostridium* and *Lactobacillus*. Consequently, babies born via cesarean section present a lower diversity and abundance of microorganisms considered beneficial compared to those born vaginally [96].

### 4.4. Type of Feeding

The type of feeding is another primary modulator of the microbiota. Breast milk provides all the initial nutritional requirements for the newborn’s rapid growth during the first few months of life, in addition to containing a wide variety of protective factors, such as immunoglobulin A, cytokines, fatty acids, oligosaccharides, lysozymes, or lactoferrin [97]. Although the composition of breast milk varies according to the mother’s lifestyle and diet [98], certain commensal bacteria (*Lactobacilli*, *Bacteroides* spp., and *Bifidobacterium* spp.) [99] have been associated with healthy milk. Furthermore, breast milk contains specific oligosaccharides that serve as prebiotics, selectively promoting the growth of *Bifidobacterium* spp. and the production of SCFAs. Indeed, breastfeeding has been associated with a reduced risk of developing allergies, asthma, obesity, and metabolic diseases in the future.

In contrast, babies fed with formula milk show a more diverse microbiota similar to that of adults, with higher proportions of *Bacteroides* spp., *Clostridium* spp., and a lesser predominance of *Bifidobacterium* spp. Although greater microbial diversity is generally considered beneficial in adults, in babies, a microbiota dominated by *Bifidobacterium* spp. associated with breastfeeding is deemed optimal for the early development of the immune and metabolic system [100].

### 4.5. Antibiotic Exposure

Exposure to antibiotics is another potential modulator of the microbiota described [101]. This exposure can affect the fetus’s microbiota, whether it occurs during pregnancy, at the moment of delivery, or during the breastfeeding period. Antibiotics can alter the composition and diversity of the maternal microbiota, including vaginal, intestinal, breast milk, and potentially placental microbiota [102]. These changes can influence the microbiota that the fetus encounters and acquires during delivery and birth, potentially resulting in a reduced or altered initial colonization of beneficial bacteria such as *Bifidobacterium* spp. and *Lactobacillus* spp., which are important for the development of the baby’s immune system and protection against pathogens [103].

### 4.6. Environmental and Genetic Influences

The influence of genetics on the human resident microbiota has been demonstrated, although the mechanisms remain elusive. A recent study in mice has linked specific bacterial strains to particular chromosomes, significantly advancing our understanding of how genetics shapes microbiota development. Additionally, the substantial impact of environmental factors has been consistently observed in various previous studies, underscoring their role alongside genetics in influencing microbiota composition [104,105]. Specifically, among the environmental factors that most influence this early stage of life, environmental pollution and the presence of siblings and pets stand out [106,107].

The geographic area of residence will be determinant in this environmental exposure, varying considerably between urban and rural areas [108]. In environments with significant air pollution, a depletion of Firmicutes, especially of the genus *Bifidobacterium*, has been detected, which is associated with inflammatory diseases [109].

Regarding living with siblings, the study by Talavoire et al. [110] demonstrated that siblings living in the same house shared a greater similarity of microbiota, even if they were not genetically related. This cohabitation with siblings has been linked to the development of a more diverse microbiota in the early years of life [111]. Specifically, Christensen et al. [112] found that children with older siblings (regardless of the number) exhibited a lower abundance of *Veillonella* spp. and *Enterobacteriaceae* such as *Escherichia* spp. and *Shigella* spp., along with a higher abundance of *Prevotella* spp. This fact was associated with a reduced risk of developing atopic diseases and asthma during the early stage of life.

Regarding exposure to animals, evidence has been found that direct and prolonged contact shapes the microbiota. In the study by Song et al. [113], adults cohabiting with a dog not only showed similar microbiota among themselves but also exhibited overlap in microbial communities with their dog. This exposure has been associated with a lower predisposition to allergies in the future [114].

### 4.7. Microbiota Stabilization

Over time, the initial facultative anaerobic colonizers are gradually displaced by conventional anaerobic bacteria. When the child is weaned or when solid foods are introduced, their microbiota begins to resemble that of adults, with an increase in the number and variety of bacterial species and strains; they harbor around 500 to 1000 species, mainly bacteria of the phyla *Bacteroidetes* (25%) and *Firmicutes* (60%) [115].

This intestinal microbiota eventually achieves a state of maturity similar to that of an adult, approximately by three years of age [116]. During this period, the microbiota undergoes further diversification and becomes more stable.

## 5. Conclusions

Studies on the microbiota have expanded in recent years, evolving from merely identifying the presence and quantity of specific microbial communities to understanding the production and modification of metabolites by these communities.

These metabolites are indicators of the bacterial activity occurring at any given time. Thus, understanding the mechanisms leading to their production will allow us to comprehend what is happening and how to modulate their effects according to our interests. We have observed that the range of metabolites is broad, serving various functions. They have been linked to gastrointestinal diseases such as Crohn’s disease, ulcerative colitis, irritable bowel syndrome, and colorectal cancer; metabolic diseases including obesity, type II diabetes, and dyslipidemias; autoimmune diseases such as rheumatoid arthritis, allergies, and asthma; neurodegenerative diseases like Alzheimer’s and Parkinson’s; depression and anxiety; hypertension and other cardiovascular diseases; and skin diseases such as acne, eczema, and psoriasis.

All these conditions share the development of an uncontrolled inflammatory state. However, the metabolites produced, as well as the microbiota involved, must be appropriately selected for each study and disease involved. There remains a long way to go in understanding how this microbiota affects our health. More studies are needed that provide as complete a view as possible of all the interactions that occur in this complex scenario.

It should be noted that the microbial community is not composed only of bacteria. Fungi, viruses, and archaea can also play significant roles, despite their scarce presence compared to bacteria. Additionally, it is important to consider that the microbial composition varies depending on the location studied, which will affect the type and quantity of functional products produced by it.

In summary, this is a challenging yet promising field of study where biomarkers and therapeutic targets can be identified for both healthy microbiota and states of dysbiosis that may prevent future diseases. Since the establishment of the microbiota occurs in the early years of life, studies should be aimed at understanding these initial interactions that will shape our future microbial trajectory.

## Data Availability

The original contributions presented in the study are included in the article, further inquiries can be directed to the corresponding authors.
